# Pneumatosis Cystoides Intestinalis Mimicking Perforation in a Patient With Gastric Signet Ring Cell Carcinoma: A Case Report

**DOI:** 10.1002/ccr3.72233

**Published:** 2026-03-17

**Authors:** Yongshuai Lin, Weimin Wu, Xin Zhao, Jun Wang, Juan Yang

**Affiliations:** ^1^ Department of General Surgery The 928th Hospital of the Joint Logistics Support Force, CPLA Haikou Hainan China

**Keywords:** case report, gastric signet ring cell carcinoma, gastrointestinal perforation, misdiagnosed, pneumatosis cystoides intestinalis

## Abstract

Pneumatosis cystoides intestinalis (PCI) poses a diagnostic challenge by radiographically mimicking surgical emergencies like perforation. This case highlights that while PCI itself may be benign, the decision for exploration must weigh the risk of missing a life‐threatening condition or a coincident malignancy.

## 1 Background

Pneumatosis cystoides intestinalis (PCI) is a relatively rare clinical condition that may arise from damage to the intestinal barrier or increased intraluminal pressure, resulting in the penetration of gas through the intestinal wall and its subsequent accumulation within the wall layers. The gas may also penetrate the wall into the abdominal cavity or accumulate within the mesentery [1]. Although PCI itself does not always present with significant symptoms, in certain cases, it is indicative of more severe underlying gastrointestinal conditions, such as intestinal perforation, obstruction, or ischemic bowel disease [2, 3, 4]. Owing to its rarity, PCI is often difficult to diagnose clinically, leading to potential delays in diagnosis and treatment. In cases of gastric cancer, especially signet ring cell carcinoma (SRCC) which is known for its aggressive behavior and poor prognosis, current guidelines emphasize the importance of accurate staging and multidisciplinary management, which may include perioperative chemotherapy for locally advanced disease [5, 6]. This study presents a clinical case of PCI that was initially misdiagnosed as gastrointestinal perforation complicated by gastric signet ring cell carcinoma and discusses its diagnostic and therapeutic management.

## 2 Case Presentation

A 55‐year‐old man was admitted to the nephrology department in December 2024 with acute‐onset generalized weakness and confusion that began approximately 7 h prior. He reported a history of recurrent abdominal distension and acid reflux for over a month, particularly within half an hour after meals. About 10 days before admission, these symptoms worsened and were accompanied by nausea and vomiting. The vomitus consisted of gastric contents without evidence of blood (hematemesis). He self‐administered over‐the‐counter omeprazole without medical consultation, which provided only temporary relief. He had a 30‐year smoking history (half to one pack per day) and reported occasional alcohol consumption. There was no significant history of trauma, prior surgery, or drug allergies, and no known family history of gastric cancer or other malignancies. His body mass index (BMI) was 18.3 kg/m^2^, and he reported an unintentional weight loss of approximately 5 kg over the preceding month. Upon admission, the patient's vital signs were as follows: temperature, 36.5°C; pulse, 89 beats/min; respiratory rate, 20 breaths/min; and blood pressure, 170/92 mmHg. The patient appeared mildly lethargic, with poor nutritional status. His mental status was somewhat impaired, he exhibited speech disturbance, and he was partially uncooperative during the physical examination. Cardiopulmonary examination was unremarkable. The abdomen was soft, with no obvious tenderness or rebound tenderness. Bowel sounds were auscultated at 4–5 per minute. The liver and spleen were not palpated below the costal margin, and there was no percussion pain in the liver or kidney regions. The patient exhibited generalized weakness and was unable to walk, but there was no lower extremity edema. Laboratory investigations revealed leukocytosis (white blood cell count 17.55 × 10^9^/L) with neutrophilia (91.0%), elevated creatinine (423 μmol/L) and blood urea nitrogen (28.48 mmol/L), indicative of acute kidney injury. Arterial blood gas analysis showed severe hypokalemia (2.67 mmol/L), hyponatremia (124.1 mmol/L), hypochloremia (44.6 mmol/L), and metabolic alkalosis (pH 7.57, ${\mathrm{HCO}}_{3}^{-}$ 29.3 mmol/L).

**FIGURE 1:**
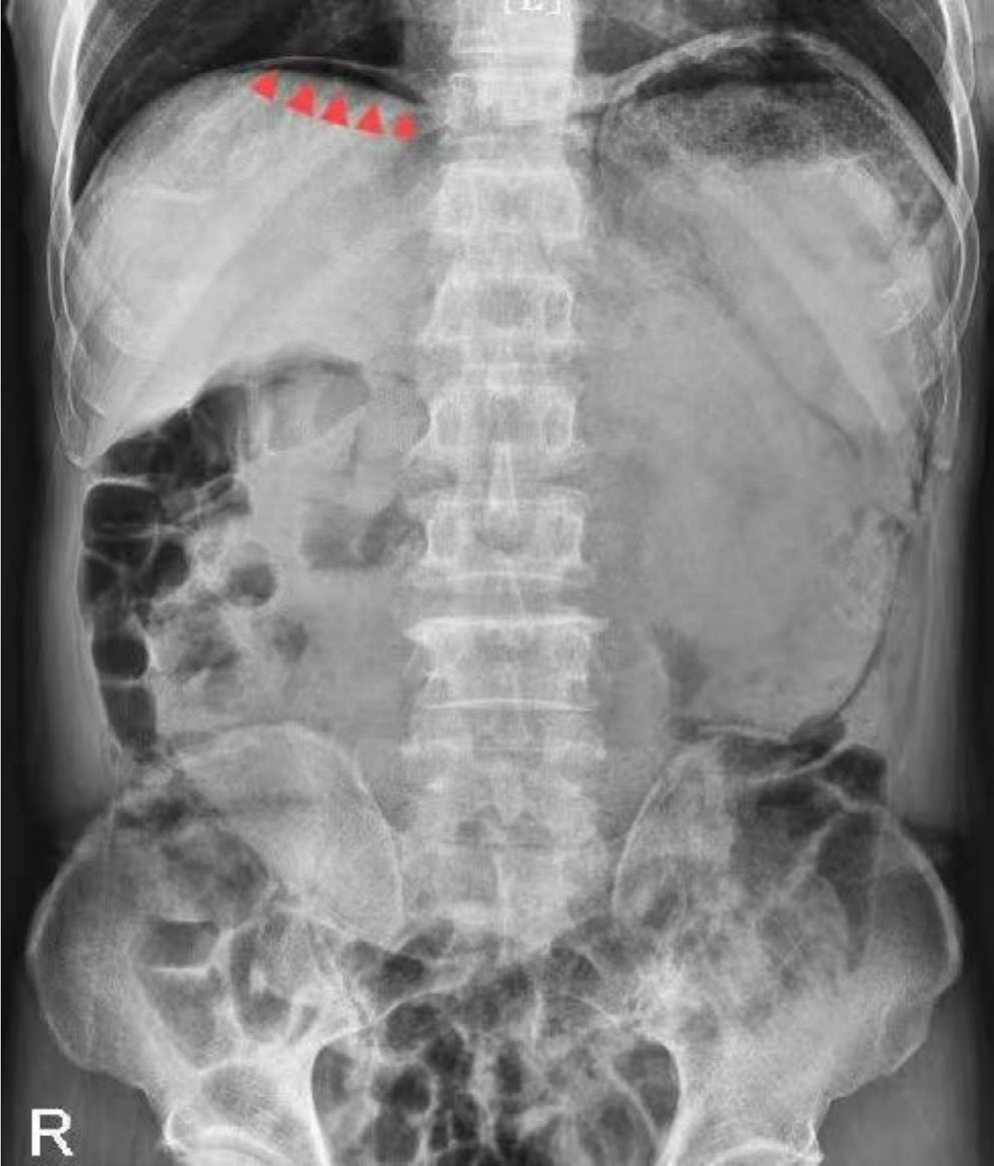
An upright abdominal DR image revealed the presence of free gas beneath the diaphragm.

**FIGURE 2:**
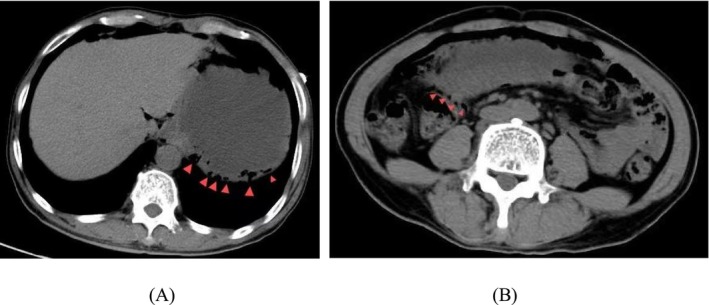
Abdominal CT imaging upon hospital admission. (A) Abdominal CT revealed scattered submucosal free gas within the gastric wall. (B) Multiple cystic changes beneath the serosa of erathered intestinal walls, resembling a cluster of grapes appearance.

**FIGURE 3:**
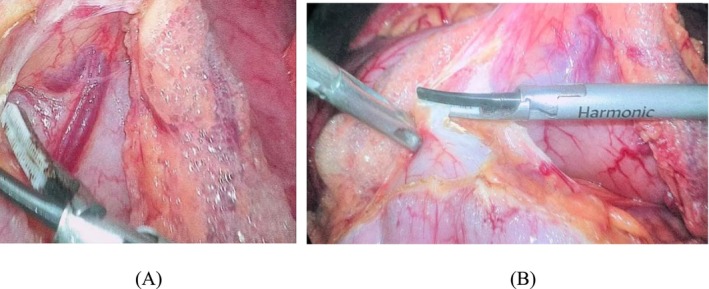
Intraoperative findings revealed bubble‐like changes in portions of the gastric greater omentum and transverse mesocolon. (A) PCI is identified in the gastric greater omentum. (B) The PCI is identified in the transverse mesocolon.

The patient was actively treated for acute renal failure, and the electrolyte imbalance was corrected after admission. Subsequent upright X‐ray of the abdomen revealed a small amount of free air beneath the right diaphragm, suggesting gastrointestinal perforation (Figure 1). Further abdominal CT examination revealed a space‐occupying lesion in the gastric antrum with secondary obstruction, resulting in gastric content retention, scattered submucosal gas in the gastric wall, and free air in the peritoneal cavity, strongly indicating the possibility of gastric wall perforation. Additionally, a low‐density lesion was noted in the left lateral lobe of the liver (Figure 2). After consultation in our department, gastric cancer complicated by obstruction leading to gastrointestinal perforation was suspected. On December 6, 2024, the patient was transferred to our department. Following the exclusion of any absolute surgical contraindications, emergency laparoscopic exploration was arranged under general anesthesia. During the procedure, a malignant tumor approximately 3 cm × 3 cm in size was identified in the gastric antrum, invading the serosal layer, but no perforation was found. The anterior and posterior walls of the stomach and the duodenal wall appeared smooth, with no significant edema or perforation. Further exploration of the jejunum, ileum, cecum, ascending colon, transverse colon, descending colon, and sigmoid colon revealed no signs of rupture or perforation. Notably, intraoperative exploration revealed bubble‐like changes in the greater omentum and transverse mesocolon (Figure 3), suggesting the presence of pneumatosis cystoides intestinalis. According to the exploration results, the intraoperative diagnosis was as follows: 1. Malignant tumor in the gastric antrum; 2. Pneumatosis cystoides intestinalis. Laparoscopic radical gastrectomy for gastric cancer was then performed.

Postoperative Course and Recovery (Table 1): The patient fasted for 6 days postoperatively, receiving anti‐infective therapy, parenteral nutritional support, and symptomatic management. The pelvic and abdominal drainage tubes were removed on postoperative days 8 and 10, respectively. His clinical condition steadily improved, and he was discharged in good condition on postoperative day 12. At the 1‐month follow‐up, he had recovered to a near‐normal state and could engage in moderate physical activity.

**TABLE 1:** Clinical timeline of key events.

Date/Time point	Event
> 1 month before admission	Onset of recurrent abdominal distension and acid reflux
~10 days before admission	Symptom exacerbation with nausea and vomiting
~7 h before admission	Acute onset of limb weakness and confusion
December 4, 2024	Admission to Nephrology. Imaging revealed pneumoperitoneum and gastric mass
December 6, 2024	Transfer to General Surgery; emergency laparoscopic exploration and radical distal gastrectomy performed
December 13, 2024	Pelvic drain removed. Final pathology report obtained
December 15, 2024	Abdominal drain removed
December 17, 2024	Discharge from hospital
~2 weeks post‐discharge	Commenced adjuvant therapy (SOX + Sintilimab)
One‐month follow‐up after discharge	The patient had recovered to a near‐normal state and could engage in moderate physical activity

**FIGURE 4:**
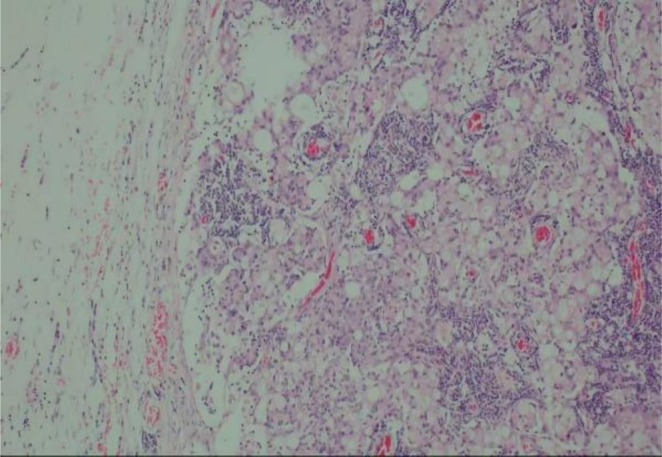
Hematoxylin and eosin (HE) staining revealed infiltrative growth of tumor cells with a signet‐ring morphology (×100).

Pathological Findings and Adjuvant Therapy: The definitive pathological report (December 13, 2024; Figure 4) confirmed infiltrative signet‐ring cell carcinoma of the gastric antrum (ulcerative pattern), measuring 6 × 4 × 4 cm, with invasion into the serosal layer (pT4a). Metastatic carcinoma was present in the lesser curvature (6/6), greater curvature (8/8), and group 9 (3/3) lymph nodes, with all margins negative, yielding a final pathological stage of pT4aN3bM0 (Stage IIIB, AJCC 8th edition). Immunohistochemistry was positive for CDX‐2, CEA, and CK, with proficient mismatch repair (MLH1+, MSH2+, MSH6+). A postoperative contrast‐enhanced abdominal CT scan suggested the previously noted left hepatic lobe low‐density lesion was more consistent with a hemangioma. Based on this high‐risk pathology (pT4aN3b), the patient was referred to medical oncology and commenced adjuvant combination therapy with the SOX regimen (oxaliplatin plus tegafur) combined with sintilimab (an anti‐PD‐1 antibody) in the second week after discharge.

## 3 Discussion and Conclusions

Pneumatosis cystoides intestinalis is an exceptionally rare disease characterized by the formation of varying numbers and sizes of gas‐filled cysts within the intestinal wall. These cysts can be distributed throughout the gastrointestinal tract, with the subserosal or submucosal layers of the small intestine and colon being the most commonly affected sites [7]. It was first discovered and documented by DuVernoi in 1730 during an autopsy [8]. However, Lerner and Gazin did not achieve the first radiological diagnosis of the condition until 1946 [9]. Since then, with advancements in auxiliary diagnostic techniques, reports of PCI have gradually increased. Jamart, in a study involving 919 patients, reported that the peak incidence of the disease occurs between the ages of 41 and 50, with a male‐to‐female ratio of 3:1 [10].

The pathophysiology and etiology of PCI remain unclear. Currently, three hypotheses are widely recognized as potential mechanisms of disease development: the mechanical theory, the pulmonary theory, and the bacterial theory [9, 11, 12]. The mechanical theory suggests that gas enters the intestinal wall through damaged gastrointestinal mucosa and spreads along tissue planes to the submucosal or subserosal layers. However, while mucosal injuries are relatively common, PCI remains exceedingly rare. Furthermore, this hypothesis has not been substantiated by animal experiments. The pulmonary theory posits that gas originates from ruptured alveoli, migrates into the mediastinum, and subsequently travels along the perivascular spaces of the aorta and mesenteric vessels to reach the mesentery, gastrocolic ligament, and subserosa of the intestinal wall. Pulmonary diseases such as asthma, interstitial pneumonia, and chronic obstructive pulmonary disease (COPD) can cause alveolar rupture, leading to the release of gas into mesenteric vessels. However, the majority of patients with pneumatosis cystoides intestinalis do not present with associated pulmonary conditions. The bacterial theory suggests that PCI results from infection of the intestinal lymphatic vessels by gas‐producing bacteria. Reports have documented the isolation of *Clostridium perfringens* from cysts in such cases. Moreover, animal experiments have demonstrated that injecting *C. perfringens* into the abdominal cavity or intestinal wall can induce a model of pneumatosis cystoides intestinalis [13, 14]. Furthermore, some studies suggest that treating bacterial overgrowth with metronidazole can alleviate this condition [15, 16]. In this case, the patient was treated postoperatively with a combination of metronidazole and third‐generation cephalosporins. Compared with admission findings, follow‐up CT scans revealed a reduction in free intraperitoneal gas after infection control, providing further support for this theory.

Reports of PCI have also been documented in patients with certain autoimmune diseases and diabetes, although the underlying etiology remains poorly understood [17, 18] These occurrences may be related to the pharmacological regimens administered in these populations. Recent studies have revealed a potential association between the therapeutic use of α‐glucosidase inhibitors and the occurrence of PCI. The mechanism is thought to involve intestinal gas production through fermentation by the intestinal flora of carbohydrates, whose absorption is inhibited by α‐glucosidase inhibitors [19].

PCIs can affect the entire gastrointestinal tract, from the esophagus to the rectum, with the small intestine and colon being the most commonly involved sites. The left colon is the most frequently affected area of the colon [20]. In addition, PCI can occur in the mesentery, omentum, hepatogastric ligament, or other regions [20, 21]. In this case, the involvement was primarily in the mesentery and omentum, presenting as multiple gas cyst‐like changes in the affected area. PCIs typically exhibit nonspecific clinical symptoms and may be asymptomatic or present with complaints such as abdominal pain, bloating, diarrhea, nausea, vomiting, or gastrointestinal bleeding [22, 23]. When PCI involves the stomach, duodenum, or small intestine, symptoms such as nausea, vomiting, and bloating are common. Moreover, PCI affecting the colon often manifests as melena or diarrhea. In rare cases, severe acute abdominal conditions, such as intestinal obstruction or perforation, may occur [24]. In this case, the patient presented to our hospital with limb weakness and incoherent speech, accompanied by nausea, vomiting, and abdominal bloating during the course of illness. We hypothesize that severe vomiting may have resulted in hypokalemia, hypochloremia, and metabolic alkalosis, leading to the observed limb weakness and slurred speech. This hypothesis is supported by the patient's electrolyte, pH, and HCO3^−^ measurements obtained after admission. Importantly, the patient had an underlying gastric malignancy. Therefore, we cannot rule out the possibility that nausea, vomiting, and abdominal bloating were caused by tumor‐related gastric obstruction or a combination of both factors.

At present, the diagnosis of PCI is primarily based on CT scans, X‐ray, colonoscopy, and endoscopic ultrasound (EUS) examinations. CT scans can be performed without or with intravenous contrast agent injection. The typical CT scan features of PCI include grape‐like clusters or honeycomb‐shaped shadows on the intestinal wall, multiple gas shadows around the intestine, and free intraperitoneal gas [25]. Abdominal X‐rays may reveal subdiaphragmatic free gas or multiple small gas cysts on the intestinal wall [26]. The most intuitive examination for PCI is colonoscopy, which typically reveals multiple circular or irregularly shaped air bubbles or sacs of varying sizes on the intestinal wall. These structures are soft to the touch and can deform under pressure from biopsy forceps. Upon puncture, the gas‐filled cysts collapse [27]. Additionally, EUS is often considered the diagnostic modality of choice for reliably identifying PCI. It typically reveals multiple irregular hyperechoic structures with posterior acoustic shadowing located within the submucosa or subserosa [28, 29].

On the basis of the characteristics of onset, PCIs can be classified into primary or idiopathic (15%) and secondary (85%) types [20, 25]. Primary or idiopathic PCI, as well as most secondary cases, are typically asymptomatic or present with only mild abdominal discomfort, often being diagnosed incidentally through radiological imaging [22, 26]. These patients generally do not require specialized treatment and can be managed with conservative approaches, including antibiotics (cephalosporins and metronidazole), hyperbaric oxygen therapy, dietary modifications, and endoscopic interventions [15, 27]. Studies have shown that approximately 70% of patients experience relief through nonsurgical treatments [17]. However, a small number of patients with secondary PCI usually have severe symptoms or acute abdomen, which often require surgical intervention [2, 4]. In particular, for patients with adverse prognostic factors such as obstruction symptoms, a significant increase in white blood cells, acute kidney function injury, or hypotension, laparotomy or laparoscopic exploration surgery should be performed immediately after sufficient preoperative preparation [30, 31, 32].

Signet ring cell carcinoma is a distinct histologic subtype of gastric cancer characterized by intracellular mucin accumulation displacing the nucleus [33, 34]. It often presents at a more advanced stage and is associated with a potentially poorer prognosis compared to other adenocarcinoma subtypes [35, 36]. Management follows the general principles for gastric adenocarcinoma, with surgical resection being the cornerstone of curative intent treatment for localized disease. For locally advanced cases (e.g., T3/T4 or node‐positive), current guidelines strongly recommend perioperative chemotherapy (e.g., FLOT regimen: fluorouracil, leucovorin, oxaliplatin, and docetaxel) based on improved survival outcomes demonstrated in clinical trials [5, 6, 37, 38]. Interestingly, in our case, the patient developed significant renal failure, and subsequent radiological findings suggested a severe condition. However, the patient's abdominal symptoms and signs were not as pronounced; the patient presented only with nausea, vomiting, and abdominal bloating, without any significant abdominal tenderness or other notable signs. An important consideration in this case is the impact of the presumed emergency (suspected perforation) on the therapeutic pathway for the underlying gastric cancer.

Under elective circumstances, a patient with locally advanced, obstructive gastric cancer would typically undergo staging, nutritional optimization, and potentially neoadjuvant chemotherapy before surgery [5, 6, 37]. The presence of pneumoperitoneum, a classic sign of perforation, understandably altered this trajectory, necessitating immediate surgical exploration. This highlights a critical diagnostic dilemma where a benign condition (PCI with pneumoperitoneum) can compel management that diverges from the optimal planned approach for a concurrent malignancy. In the present case, the emergent surgical intervention was prompted by the suspicion of perforation. While this precluded standard preoperative chemotherapy, the decision for immediate exploration was justified given the life‐threatening differential diagnosis [39, 40]. Postoperative adjuvant therapy was planned in accordance with the pathological findings and guideline recommendations [5, 6].

The strength of this report lies in the detailed documentation of a rare clinical mimicry, where PCI with pneumoperitoneum masqueraded as a surgical emergency in a patient with an advanced, yet previously undiagnosed, gastric malignancy. The use of laparoscopy allowed for definitive diagnosis and oncologic resection. The main limitations are inherent to the case report format, including its single‐patient focus and the inability to assess long‐term survival outcomes, particularly in the context of emergent versus electively sequenced therapy.

In conclusion, PCI is an extremely rare condition, and its coexistence with gastric signet ring cell carcinoma is even rarer. This is especially true when radiological findings suggest free intraperitoneal gas, but abdominal symptoms and signs do not support a diagnosis of gastrointestinal perforation. In such cases, it is important to consider the patient's overall clinical situation and maintain a high level of suspicion for PCI. By summarizing the potential causes, clinical manifestations, imaging features, endoscopic findings, and treatment approaches for PCI patients, our study aims to increase awareness of this condition and provide valuable insights for clinical diagnosis and management.

## Author Contributions

**Yongshuai Lin:** conceptualization, visualization, writing – original draft, writing – review and editing. **Weimin Wu:** investigation, resources. **Xin Zhao:** data curation, investigation, resources. **Jun Wang:** supervision, writing – review and editing. **Juan Yang:** project administration, supervision, writing – review and editing.

## Funding

This case report was not supported by relevant funds.

## Ethics Statement

The authors have nothing to report.

## Consent

Written informed consent was obtained from the patient for the publication of this case report and any accompanying images.

## Conflicts of Interest

The authors declare no conflicts of interest.

## Data Availability

The data that support the findings of this study are available on request from the corresponding author. The data are not publicly available due to privacy or ethical restrictions.
